# Melanoma and the Gastrointestinal (GI) Tract: Maintaining a High Index of Suspicion

**DOI:** 10.7759/cureus.13408

**Published:** 2021-02-18

**Authors:** Hassnain R Syed, Suman Shekar, Avinash Aravantagi

**Affiliations:** 1 Internal Medicine, University of Kentucky, Bowling Green, USA; 2 Gastroenterology, Medical Center at Bowling Green, Bowling Green, USA

**Keywords:** melanoma, duodenum, stomach, metastatic

## Abstract

Malignant melanoma is a life-threatening malignant tumor deriving from melanocytes, regarded as the most lethal form of skin cancer. One of the attributing factors to this fact is its propensity to metastasize to all organs of the human body. The strongest risk factors for melanoma include exposure to UV rays, family history of melanoma, and a prior history of melanoma. Malignant melanoma is thought to metastasize first to the local lymph nodes and then to secondary sites, most commonly skin, lung, and to the brain. This case highlights the severity of melanoma and its negative impact on the gastrointestinal tract. Patients with metastatic melanoma to the gastrointestinal tract can present with nonspecific, generalized gastrointestinal symptoms such as abdominal pain or constipation. Here we discuss the pathology, symptomatology, management options, and prognosis of metastatic melanoma of the gastrointestinal tract. The aim of this case is to promote a high index of suspicion of gastrointestinal metastasis in melanoma patients with gastrointestinal symptoms.

## Introduction

Malignant melanoma is the fifth most common cancer among the population, and it is known to spread to most organs in the body [1]. Severity of this dastardly disease is predicated based on the stage of the cancer. Melanoma can be divided into five stages, stage 0 - stage IV, where patients with stage II to IV disease are more likely to develop metastatic disease [1]. It is one of the most common cancers that can disseminate to the gastrointestinal tract (GIT). In an analysis of 652 patients with metastatic melanoma performed by Gupta and Brasfield, the incidence of metastasis within the GIT was shown to be: Liver 68%, Jejunum and ileum 58%, stomach 26%, colon 22%, duodenum 12%, rectum 5%, and anus 1% [2]. This case report aims to add yet another example of this phenomenon to the literature. Here, we present a 49-year-old female with biopsy proven melanoma of the back, who presented three years later with generalized gastrointestinal symptoms, and was found to have metastasis to the stomach and duodenum.

## Case presentation

**Figure 1:**
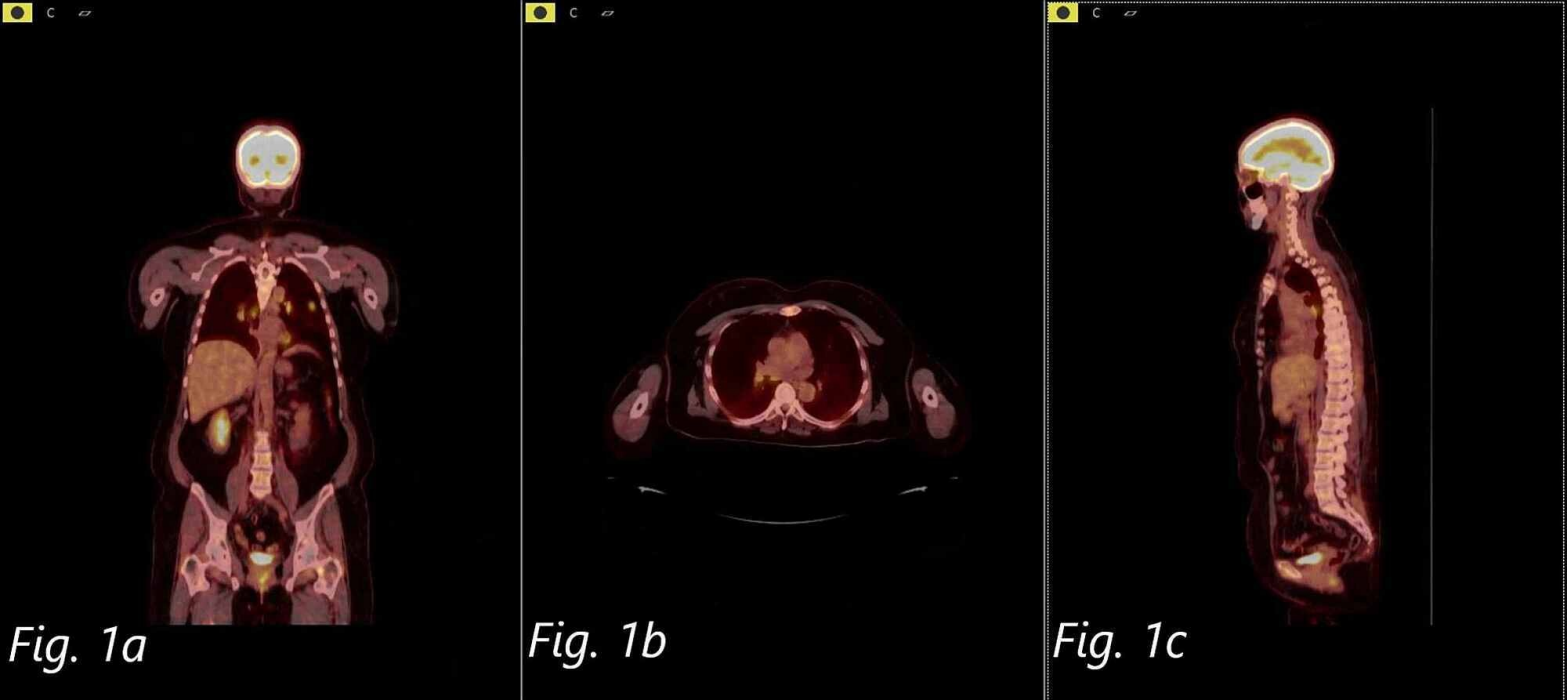
Coronal (a), axial (b), and sagittal (c) views of FDG PET/CT scan whole body showing multiple areas of hypermetabolic activity indicative of metastasis. FDG: Fluorodeoxyglucose; PET: Positron emission tomography.

Our patient is a 49-year-old female who presented with chief complaints of decreased oral intake, generalized abdominal pain, fatigue, a 30-pound weight loss, with nausea and vomiting for eight weeks. She was diagnosed with superficial spreading melanoma of the back (depth of 2.85 mm and Clark's level III) three years prior. Dermatopathology showed predominant lymphocytic response with epithelioid melanocytes with variable melanin pigment deposition. The tumor was excised, and right axillary sentinel lymph node biopsy was also performed at that time which was negative for metastasis. Hence the tumor was staged at stage II, T3aN0M0. Two years post-surgery she had a screening CT chest which showed pulmonary nodules and enlarged lymph nodes suggestive of metastatic disease (stage IV), and she was subsequently started on immunotherapy with nivolumab. A follow-up PET/CT scan revealed increased metabolic activity in the lungs, several hypermetabolic foci in the liver, increased metabolic activity in the right paraspinous muscles, and bone lesions in the left hip, left transverse processes of L5, left L3 vertebral body, and in the sternum-all suggesting metastatic disease (Figure 1a-1c).

At present, review of systems was remarkable only for generalized abdominal pain. She denied dizziness, confusion, recent falls, or difficulty ambulating. She reported intact gross and fine motor skills. Physical exam revealed a large scar on the posterior back with keloid formation. Cardiovascular and respiratory exams were unremarkable.

**Figure 2:**
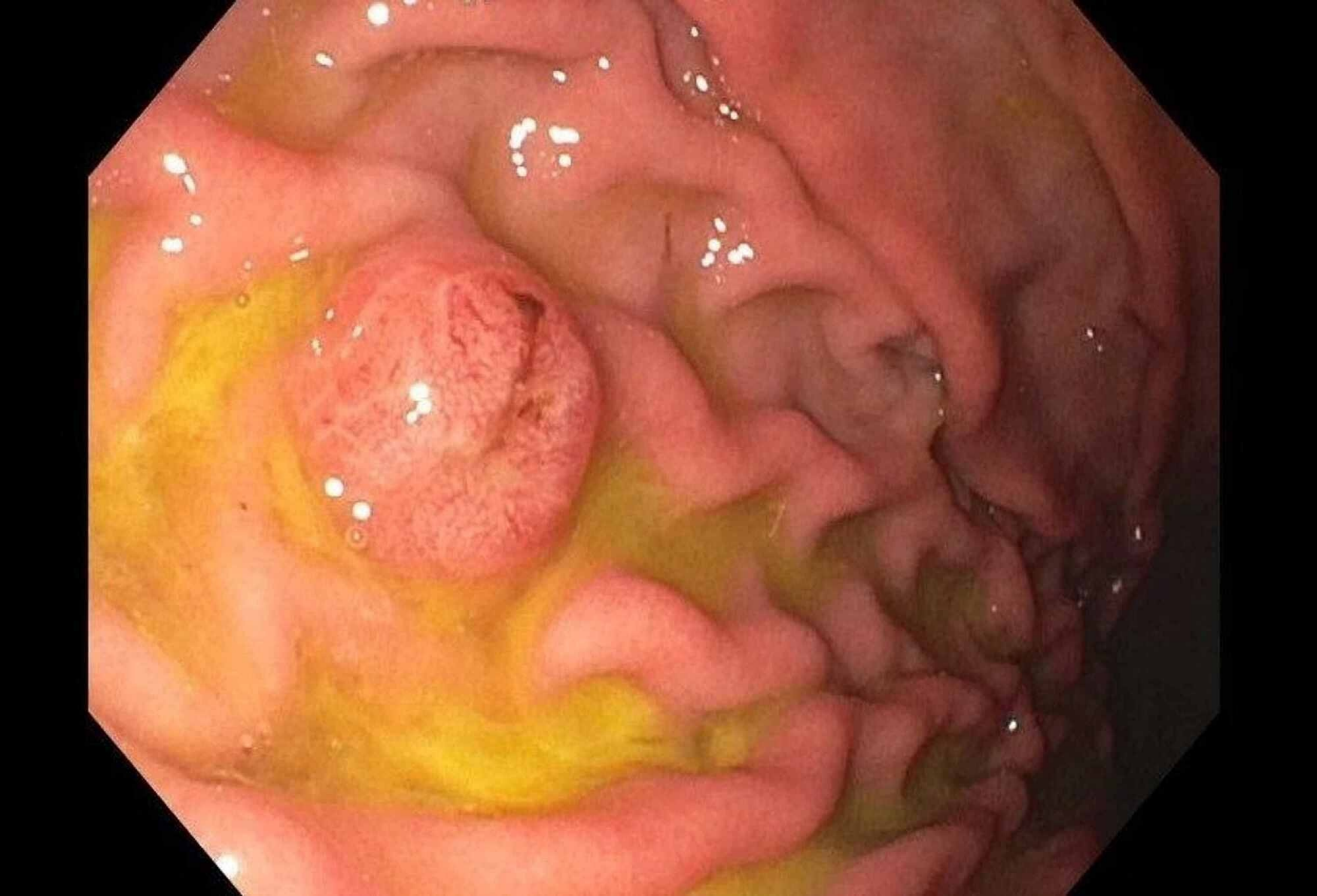
Mucosal nodule seen in gastric body.

**Figure 3:**
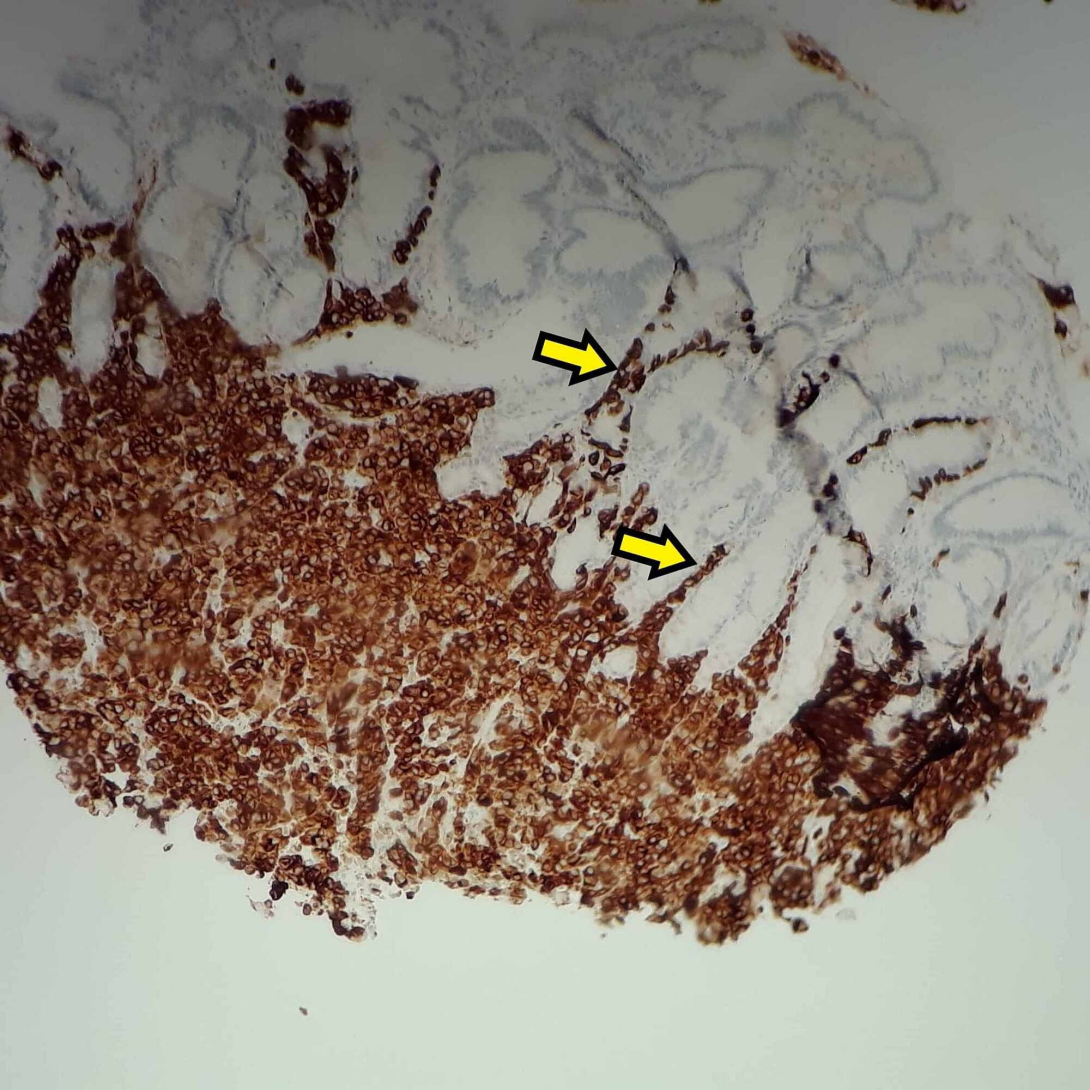
Gastric nodule seen at 100x, stained MART-1 positive (brown). Metastatic infiltration of glands seen (yellow arrows).

**Figure 4:**
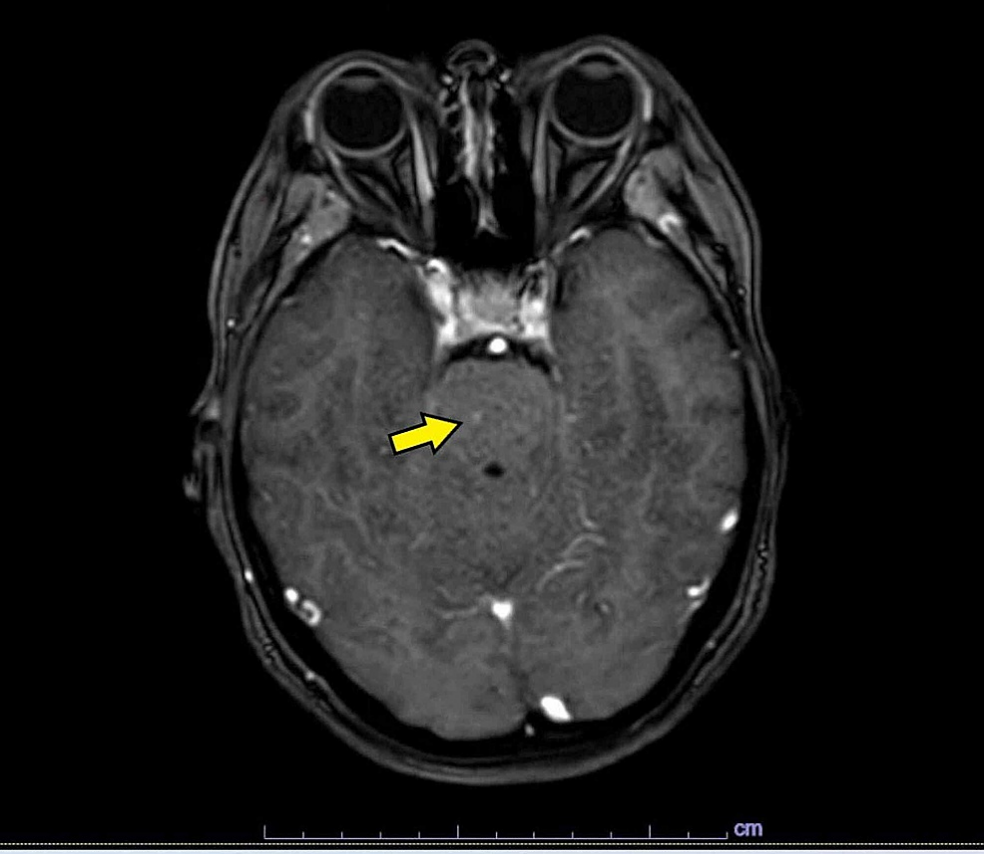
MRI brain. Gadolinium-enhanced T1-weighted axial image showing punctate focus of enhancement at right pons (yellow arrow).

Given patient's deteriorating functional status and failing nutritional requirements, it was decided to perform a percutaneous endoscopic gastrostomy. This revealed three mucosal polypoid nodules, approximately 5-10 mm with tip ulcerations, in the gastric body and multiple mucosal nodules, approximately 5-10 mm, in the duodenum (Figure 2). Biopsies of these nodules showed S-100 and MART-1 positive metastatic melanoma (Figure 3). Further diagnostic studies included MRI of the brain with and without contrast, which showed a very subtle region of increased signal in the right pons (Figure 4), with a punctate focus of enhancement measuring 1 mm in size. Genetic sequencing revealed mutations in the following genes: BRAF V600E, PTEN, PD-L1 28-8.

The patient was started on enteral nutrition with a goal rate of 70 ml/hour. Nausea and vomiting were managed with combination of antiemetics. She was referred to radiation oncology for stereotactic radiosurgery treatments. The patient was also referred for the Sarah Cannon clinical trial research program for further management of her metastatic melanoma.

## Discussion

Metastatic melanoma of the GIT is a common postmortem finding, however antemortem diagnosis of this lethal disease is quite rare [2]. In fact, clinical diagnosis of metastatic melanoma of the GIT is made in only 2% of cases [3]. This is most likely due to the seemingly vague and general constellation of presenting symptoms once metastasis has occurred. Such symptoms include nausea, vomiting, and abdominal pain. Moreover, the situation becomes more complicated when considering that melanoma in the GIT can also be a primary cancer.

There are four major types of cutaneous melanoma. In descending order of frequency, these are: superficial spreading, nodular melanoma, lentigo maligna, and acral lentiginous [4]. Histopathology is the gold standard of diagnosis of melanoma. However, the detection of immunohistochemical proteins is employed when nodal metastasis is under suspicion. The most widely used “melanoma markers” are: S-100, HMB-45, and MART-1/Melan-A [5]. It is also important to appreciate that due to their wide differences in sensitivity and specificity, these markers are often tested together to optimize statistical yield.

Interestingly enough, melanomas can also stem from mucosal epithelium which lines various tracts in the human body including the GIT, genitourinary tract, and the respiratory tract. These are referred to as primary mucosal melanomas. Many immunohistochemical studies have been performed which show the presence of HMB-45 and S100 stains, confirming the presence of melanocytes in the mucosa of these areas. Little is known about primary mucosal GIT melanomas, as they are extremely rare (accounting for approximately 1% of all melanomas), and not many studies have been performed regarding their management [6]. Primary mucosal GIT melanomas are usually discovered at an advanced presentation, and due to a lack of standardized therapy, they carry a far worse prognosis [7]. A diagnosis of primary GIT mucosal melanoma can be inferred histologically if a precursor lesion, or melanosis, is present in tissue samples [8]. In contrast, metastasis from a cutaneous melanoma is described as showing lymphocytic infiltration with melanophages.

Gastrointestinal symptoms should prompt one to explore further endoscopic investigation. The problem lies in the fact that most metastatic melanomas of the GIT occur in the jejunum and ileum. Hence, conventional esophagogastroduodenoscopy (EGD) and colonoscopy may not detect the cancer. Therefore, in the event of a negative endoscopy in a cutaneous melanoma patient, one should consider a capsule endoscopy to ensure complete examination of the GIT. Our patient did not receive a capsule endoscopy as metastatic lesions were identified on EGD, and performing one would not have changed management. Treatment of metastatic melanoma to the GI tract may include surgery, chemotherapy, immunotherapy, or participation in clinical trials. However, the immunocompromised state caused by chemotherapy may cause serious complications in patients with GI tract involvement [8]. Surgery is almost never curative, but it can offer considerable palliation. Wornom et al. reported on the outcomes of 65 patients who underwent surgical excision of 94 metastatic lesions from various sites, including the abdomen [9]. Relief of symptoms was obtained in 77 to 100 percent of patients in the various groups, with symptomatic relief in 100 percent of patients with abdominal metastases. A retrospective study performed by Gutman et al. showed that surgical resection of metastatic melanoma of the GIT was associated with a median survival of 11 months [10]. Immunotherapy is an important systemic treatment modality for metastatic melanoma. Our patient was started on nivolumab, an immune checkpoint inhibitor, which specifically targets the programmed cell death protein 1 (PD-1). Nivolumab is approved for adjuvant treatment of melanoma with involvement of lymph nodes or metastatic disease following complete resection. In fact, nivolumab has been shown to improve four-year recurrence-free survival (RFS) in resected stage IIIB to C and IV melanoma when compared to ipilimumab - this was demonstrated in the Checkmate 238 trial, with the primary endpoint being RFS [11].

## Conclusions

In conclusion, more research is warranted to make a case for endoscopic surveillance for GIT metastasis in patients with melanoma. Metastatic melanoma of the GIT carries a poor prognosis, but there are considerable options for palliation if caught early. This case aims to illustrate the value of having a lower threshold for endoscopic evaluation in melanoma patients. In this way, our hope is to ultimately preserve patient's quality of life.
